# Explainable retinal deep learning for cardiovascular risk stratification: a multiple modality analysis framework with vascular-centric interpretability and robustness

**DOI:** 10.1186/s12911-026-03516-y

**Published:** 2026-05-11

**Authors:** K. Sathya, G. Magesh

**Affiliations:** School of Computer Science Engineering and Information Systems, Vellore Institute of Technology, Vellore, India

**Keywords:** Retinal imaging, Cardiovascular risk, Explainable deep learning, Vascular biomarkers, Saliency analysis

## Abstract

Early non-invasive prediction of cardiovascular risk is an essential prerequisite for preventive medicine, especially in resource-limited settings. Retinal imaging provides unique insights into systemic vascular health; however, most existing deep learning models lack pathophysiological explainability and generalizability, which limits their clinical adoption. Furthermore, existing approaches fail to effectively integrate specific retinal biomarkers with multimodal indicators of cardiovascular conditions and are not adaptable to variations in demographics or imaging conditions. This limits their applicability in real-time environments, particularly within SDN-enabled 5G healthcare infrastructures, where explainability and reliability are essential. In this work, a comprehensive and explainable deep learning architecture is developed for retinal-based cardiovascular risk assessment. The proposed framework integrates five analytical components: (1) MARGE-Net, which incorporates clinical biomarkers with vascular topology; (2) SCCIM, which visualizes causally important retinal features; (3) HVAAT, a hierarchical transformer for modelling vessel attributes; (4) PaVSCM, which ensures anatomical plausibility across populations; and (5) ReCVD-LDM, which separates imaging artifacts from true anatomical predictors using contrastive learning. These modules collectively improve accuracy, interpretability, and robustness, while enabling compatibility with real-time deployment in Software-Defined Networking (SDN)-enabled 5G healthcare infrastructures. This is further supported by a conceptual edge–cloud architecture that enables efficient data transmission and low-latency inference. The model is validated on publicly available datasets, including MESSIDOR, AV-WIDE, and the STARE VAS subset, achieving an AUC of 0.94, with a 31% improvement in interpretability and 26% reduction in prediction errors caused by imaging artifacts. Overall, this work advances trustworthy and interpretable AI for cardiovascular risk prediction from retinal images in next-generation healthcare systems.

## Introduction

The human retina has emerged as a high-resolution non-invasive window into the microvascular system. It is thus becoming an increasingly important resource for the evaluation of systemic cardiovascular diseases. Epidemiological evidence has pointed out that certain retinal microvascular abnormalities, such as arteriolar narrowing, venular dilation, and arteriovenous nicking, correlate highly with powerful predictors of hypertension, atherosclerosis, and even certain myocardial infarction sets. This biological linkage also renders retinal imaging as a modality viable for the prediction of cardiovascular disease (CVD) risk, especially where direct cardiac imaging or invasive biomarkers are unavailable. Advances in deep learning have recently seen convolutional neural networks use attention-based architecture to extract high-level features from fundus photographs. While these methods reach competitive predictive performances [1–3], they hardly ever have clear explanation processes or model generalisability when subject to different conditions that affect the visual appearance of the input images. Such or wholly independent-create modality be sources to limit their clinical adoptions.

Clinically useful CVD risk models would ideally embody explainability, robustness, and physiological fidelity. Data-driven approaches alone tend to discover spurious correlations, not causal mechanisms, which could detract from clinical trust in the process. Such overfitting might be directed toward image artifacts; gradients of illumination; or imbalances with regard to patient demographics, while scattered variables of medical relevance, like arteriolar caliber and vascular branching angles, are ignored in process. Further, without counterfactual reasoning or attribute-level interpretation, these models provide little guidance to clinicians in understanding why a particular retinal pattern corresponds with elevated risks. Such limitations are significantly serious when employing a diagnostic system within an emerging 5G-based digital infrastructure in the healthcare settings. Models should, therefore, also be transparent and capable of interoperability with other modalities (for instance, clinical records, wearable devices) to be relevant in process.

Present a complete and explainable deep learning model for the context of retinal-centered cardiovascular risk predictions, along with five new modules to ensure accurate predictive value, anatomical explainability, pathophysiological grounding, and generalization across imaging variations. Among the many mechanisms proposed are multi-level attention mechanisms running parallel with vessel-specific graph embedding models, saliency-conditioned counterfactual reasoning, and latent space disentanglement. Bridge the distance between computational modelling and the clinical relevance sets. The proposed architecture presents predictive insights and actionable visualizations supporting reliable risk stratification, early intervention, and continuous patient monitoring within real-world clinical workflows in process. This is because it included hierarchies that organize different vessel attributes with metrics informed by vascular pathology sets.

The key novelty of this work lies in the integration of vascular topology modelling, counterfactual explainability, transformer-based vessel attribute analysis, and latent disentanglement into a unified framework for interpretable cardiovascular risk prediction from retinal images.

### Motivation & Contributions

Such traditional cardiovascular risk assessment pipelines are well founded mostly with invasive biomarkers and highly complex imaging procedures-echocardiography, coronary CT, which longitudinal data would have limited accessibility and scalability. With the availability of various retinal fundus cameras and mounting evidence linking retinal vascular health to the health of the heart, developing a non-invasive risk prediction algorithm based on retina has an even stronger rationale for the process. However, most contemporary deep learning models treat retina as a generic input image, without an understanding of what constitutes vascular physiology.

Mostly, these models focus on extracting high classification performance, but do not consider the interpretability, generalizability, or causal relevance of the extracted features. The lack of vascular-specific modelling corresponds with insufficient alignment with known medical knowledge, which translates to decreased clinical trust and, hence, fewer adoptions. In addition, existing explainability techniques such as Grad-CAM or LIME are post hoc and unstructured and provide vague saliency that does not translate into actionable insights for medical experts.

This paper challenges these drawbacks by proposing five fresh, analytically founded components into a consolidated, end-to-end deep learning architecture for cardiovascular risk prediction through retinal images.

- First, the MARGE-Net module introduces a dual-attention graph neural network that encodes the anatomical structure of retinal vasculature along with its associations with clinical biomarkers.
- Second, the SCCIM module provides causally grounded counterfactual visualizations, illustrating how changes in specific vascular regions influence cardiovascular risk predictions.
- Third, the HVAAT module hierarchically attends to vessel morphological descriptors such as diameter, tortuosity, and bifurcation angle, enabling multi-level interpretability across spatial scales.
- Fourth, the PaVSCM metric incorporates clinical priors to ensure physiologically plausible segmentation, thereby maintaining anatomical consistency across diverse datasets.
- Finally, the ReCVD-LDM component performs contrastive latent disentanglement to separate true anatomical predictors from confounding imaging artifacts such as illumination and acquisition variability.

Collectively, these modules form a robust, explainable, and generalizable framework that aligns with clinical reasoning. The proposed system is designed to be compatible with secure, real-time healthcare infrastructures, including SDN-enabled 5G environments, supporting efficient and reliable deployment. This work represents a significant step toward the integration of trustworthy AI for accessible and interpretable cardiovascular risk screening using retinal imaging.

## Review of existing models used for retinal cardiac analysis

Many studies have shown such applicability in real terms. For instance, Hu et al. [1] proved real-world feasibility about AI-based cardiovascular risk stratification with retinal photographs from primary care in Australia. At the same time, Diel et al. [2] classified clinical outcomes on the basis of profiles for retinal artery occlusion, corroborating the relevance of retinal markers in cerebrovascular risk stratification. Miao et al. [3] apply to such studies the concept of new metric-“retinal age gap”-where they postulate this metric as a biomarker for reproductive and vascular aging emphasizing the temporal congruence between retinal and systemic biological processes. Yang et al. [4] add on to this discussion by linking metabolic fingerprints with retinal pigment epithelium thickness, proving that changes in the retina structure can provide basis for individualized risk profiling regarding some metabolic diseases such as type 2 diabetes. Niu et al. [5] push this a step further and integrate genomic analysis of retinal vessel caliber in connection to microvascular pathways involving blood pressure and cardiovascular outcomes. Earlier report by Chen et al. [6] gives this finding even greater potencies as it links the same retinal changes with mortality by all causes and that of specific causes in U.S. adult cohorts.

Iteratively, Next, as per Table 1, Fu et al. [7] also investigate these vascular markers as they refer to glaucoma, thus implying some shared pathophysiological mechanisms between ocular and cardiovascular diseases. In that context, Germanese et al. [8] apply deep learning to OCT-A data in order to extract neurovascular features predictive of composite cardiovascular risk scores. Laying on these foundations, Courtie et al. [9] started extending oculomics into the field of acute infectious disease outcomes, with retinal morphology found to stratify COVID-19 patients in terms of ICU admission risk. The link between cardiovascular mortality and arterial stiffness in diabetic retinopathy patients has been recaptivated jointly by Kha et al. [10] and Widjaja et al. [11]. The link of diabetic retinopathy with cardiovascular-related increased mortality and arterial stiffness is also further contextualized through studies by Lal et al. [12] and Wang et al. [13] using ECG-gated retinal vessel calibers and associations with liver fibrosis, respectively. Meanwhile, Pushpanathan et al. [14] and Lee et al. [16] work together to enable robust stratification of hypertensive and diabetic patients using vision transformer architectures while enhancing interpretability in combination with high-resolution vascular detail. Age prediction with retinal biomarkers has also been furthered through the longitudinal biomarker learning by Yu et al. [17] sets. This cohort identifies, in fact, a significant thrust into broadening the application of retinal biomarkers to systemic and neurological disorders. For example, Günthner et al. [18] quantified measurable endothelial dysfunction in the retinal vasculature of patients undergoing hemodialysis for the process. At the same time, Yu et al. [19, 20] observed that risk models based on retinal parameters could also be used to predict occlusions caused by vein thrombosis even without direct fundus images & samples.

**Table 1:** Model’s empirical review analysis

Reference	Method	Main Objectives	Findings	Limitations
[1]	AI-based CVD risk assessment from retinal photography	Assess feasibility of AI-based CVD prediction in primary care	High feasibility and patient acceptability in real settings	Limited generalizability outside Australia
[2]	Comparative vascular risk profiling	Compare outcomes in RAO vs. amaurosis fugax	Central RAO linked with worse outcomes than AF	Small sample size
[3]	Retinal age gap via AI	Explore retinal age gap as reproductive aging biomarker	Positive correlation of age gap with reproductive aging	Population-specific findings
[4]	Metabolic fingerprinting on RPE thickness	Correlate metabolic markers with RPE thickness	RPE thickness variation reflects metabolic state	Requires high-res imaging
[5]	Genome-wide analysis of vessel caliber	Discover genetic basis of vessel caliber & BP	Identified new BP-microvascular pathways	Needs replication in diverse groups
[6]	Population study of retinal microvascular abnormalities	Relate microvascular signs with mortality	Retinal abnormalities predict multiple mortality causes	Observational; no causal inference
[7]	NHANES-based glaucoma risk via CV health score	Link CV health score with glaucoma risk	Glaucoma risk tied to CV health scores	Cross-sectional design
[8]	AI prediction using retinal OCT-A	Predict CV risk using retinal OCT-A via AI	Effective CV risk prediction using OCT-A and AI	Interpretability of features is limited
[9]	Retinal morphological stratification in COVID-19	Classify COVID-19 severity from retinal features	Retinal shape predicts ICU admission in COVID	Retrospective analysis
[10]	Risk analysis of DR with CVD mortality	Examine DR impact on CVD mortality	DR is a compounding risk for CVD mortality	Cohort confined to high-risk group
[11]	Neurovascular alteration analysis in diabetes with renal impairment	Evaluate neurovascular impairment in diabetic nephropathy	Retinal changes indicate systemic arterial stiffness	Causality not established
[12]	ECG-gated retinal vessel caliber assessment	Study pulsatile flow abnormalities in diabetes	Detected abnormal retinal pulsation in diabetics	Small and specific diabetic group
[13]	Linking liver fibrosis scores to retinal imaging	Associate liver fibrosis with retinal indicators	Retinal metrics linked to liver fibrosis stages	Fibrosis proxies not validated
[14]	Vision transformer for patient stratification	Stratify patients using retinal transformer models	Transformer improved risk stratification accuracy	Transformer models need larger data
[15]	Retinal vessel caliber & cognitive performance	Evaluate cognitive decline via vessel caliber	Narrowed vessels linked with cognitive decline	Ethnic generalization unclear
[16]	Vision transformer for metabolic syndrome	Classify metabolic syndrome from retina	High accuracy in metabolic syndrome prediction	Need for real-time validation
[17]	Cross-population study on retinal aging biomarkers	Study aging biomarkers with longitudinal DL	DL models showed strong age biomarker estimation	Cross-dataset variability issues
[18]	Retinal endothelial dysfunction in hemodialysis	Compare vessel function in hemodialysis vs. control	HD patients had reduced retinal endothelial function	Small control group
[19]	RVO risk prediction via tabular ML	Predict RVO risk using tabular models	High AUC for ML model without imaging	Lacks fundus image integration
[20]	Adiponectin & leptin correlation with retinal thickness	Correlate hormones with retinal metrics	Leptin levels correlated with retinal thickness	Single hormone markers used
[21]	Retinal vessel tortuosity in DR	Characterize tortuosity in DR	Tortuosity index increased in DR	Feature extraction subject to error
[22]	Retinal imaging in CKD & cardiac markers	Study retinal changes in CKD	Retinal changes mapped to cardiac stress markers	No multi-center validation
[23]	Deep learning for plaque segmentation	Segment plaques using DL	DL model achieved accurate segmentation	Requires high GPU resources
[24]	Generalizable retinal foundation model	Develop generalizable disease detection model	Model generalized across multiple diseases	Black-box model nature
[25]	Retinal thinning in CKD & eGFR correlation	Correlate retinal thinning with eGFR	Retinal thinning predicted eGFR decline	Need long-term eGFR tracking
[26]	DL system for silent brain infarction prediction	Detect brain infarction via retinal imaging	DL detected silent infarctions with high sensitivity	Clinical trial validation pending
[27]	Res-Unet for segmentation & CVD prediction	Segment vessels using optimization-based Res-Unet	Improved vessel detection and prediction accuracy	Custom optimizer complexity
[28]	AI-based CAN detection in diabetes	Detect CAN from retinal fundus	Model accurately classified CAN patients	Ethnic representation low
[29]	Remnant cholesterol & retinal morphology	Study RC and DR association	RC levels linked to DR severity	Lipid measurements not longitudinal
[30]	AI-based uveitis diagnosis from vasculature	AI diagnosis of ocular tuberculosis	AI detected vasculature changes in TB uveitis	Needs broader clinical testing
[31]	Review on RVO understanding	Review limitations in RVO understanding	Summarized gaps in RVO understanding	Mostly literature review
[32]	Glaucoma-CVD-mortality associations	Analyze glaucoma and mortality link	Confirmed glaucoma as a mortality predictor	Mortality confounders not isolated
[33]	Subclinical atherosclerosis in type 1 diabetes	Quantify subclinical atherosclerosis risk	Silent atherosclerosis raised mortality in T1D	Asymptomatic atherosclerosis detection is complex
[34]	Multimodal ECG-fundus fusion for CVD	Fuse ECG and retina for early CVD	ECG-retina fusion improved early CVD detection	ECG quality affects accuracy
[35]	Remnant cholesterol & thrombosis risk	Predict thrombosis risk via RC	RC predicted thrombosis better than LDL	Biomarker tracking over time missing
[36]	Treat-to-target management of arteritis	Update arteritis treatment targets	Stratification led to targeted arteritis therapy	Small trial scale
[37]	Genomics of retinal aging via DL	Estimate biological age from retina via DL	Genomics enhanced retinal age prediction	Ethnic-genomic link not validated
[38]	Omega-3 levels & DR progression	Study omega-3 and retinal vessel status	Omega-3 improved vascular health in T1D	Sample omega-3 variability
[39]	Visceral adiposity & cardiovascular events	Link visceral fat with CV outcomes	Abdominal fat linked with major CV events	CT confirmation not done
[40]	Genetic HTRA1 predictors for CAD/stroke	Assess genetic links to stroke/CAD	HTRA1 protease predicted stroke and CAD	HTRA1 proxy estimation challenges

Fathimah et al. [21] further validated the association between metabolic syndrome, vascular tortuosity, and retinal morphology. Mustafar et al. [22] correlated retinal findings to cardiac biomarkers directly among chronic kidney disease populations, while Jain et al. [23] emphasize the space segmentation of carotids from the retina as parallel imaging to monitoring atherosclerosis. The landmark study by Zhou et al. [24] presented a generalized retinal foundation model that identifies diseases across variable conditions and hence reflects the scalability of deep learning in retinal diagnostics. Farrah et al. [25] relate retinal thinning to kidney health, while Jiang et al. [26] apply retinal features to predict silent brain infarctions, steering the conversation from ophthalmic imaging into branches of neurology and nephrology. Meanwhile, B. S. et al. [27] claim that hybrid deep residual networks optimized for enhanced segmentation accuracy using nature-inspired algorithms for vessel detection will be of immense importance for cardiovascular predictions as downstream outputs. Nabrdalik et al. [28] prove an application of AI that classifies cardiac autonomic neuropathy from retinal images, thus promising clinical applicability among diabetic populations. In addition, Chen et al. [29] and Putera et al. [30] extend these models to lipid biomarkers and inflammatory eye diseases like ocular tuberculosis, respectively. With research by Dinah et al. [31] and Quaranta et al. [32], they present evidence for integrating retinal imaging with more systemic measures to observe associations between vein occlusion and mortality sets.

Sojo Vega et al. [33] claim that retinal imaging can unveil subclinical atherosclerosis that correlates with cardiovascular mortality risk in type 1 diabetic patients. Muthukumar et al. [34] explore sensor fusion using a combination of electrocardiogram and retinal image feature sets for the purpose of improved early detection of cardiovascular diseases. Work of Cai et al. [35] and El Miedany et al. [36] takes this interdisciplinary conversation further into lipid metabolism and inflammatory arteritis, respectively. Specifically, Huang et al. [37] provide genomic determinants for retinal aging contributing to biomarker development for aging risk in cardiovascular diseases. Newly reported biochemical and nutritional associations by Sala Vila et al. [38] accentuate the role of omega-3 on diabetic retinopathy advancement as linked via vascular status. Ueyama et al. [39] associate visceral adiposity with acute coronary syndrome patients by linking abdominal imaging biomarkers with cardiovascular outcomes, while Malik et al. [40] update on proteomic predictors for ischemic stroke, thus connecting HTRA1 protease level and coronary artery disease through retinal observations. These studies collectively represent an emerging and fast-maturing interdisciplinary field, wherein retinal biomarkers, deep learning, genomics, and systemic health indicators coalesce for early prediction, risk stratification, and clinical decision-making support in cardiovascular medicine sets.

### Retinal biomarkers and systemic associations

Advances in diabetic retinopathy research increasingly highlight links between retinal changes and systemic vascular health. Ueyama et al. [39] associated visceral adiposity with acute coronary syndrome by connecting abdominal imaging biomarkers to cardiovascular outcomes, while Malik et al. [40] provided an update on proteomic predictors for ischemic stroke, linking HTRA1 protease levels to coronary artery disease through retinal observations. These studies collectively illustrate an emerging interdisciplinary field in which retinal biomarkers, deep learning, genomics, and systemic health indicators converge for early prediction, risk stratification, and support for clinical decision-making in cardiovascular medicine. Despite these advances, existing approaches are limited by poor interpretability, insufficient multimodal integration, and reduced robustness, motivating the development of the proposed framework.

Despite these advancements, several limitations remain in existing studies. Many approaches rely on deep learning models with limited interpretability and lack integration of anatomical consistency or causal reasoning. Additionally, most methods focus on single-task prediction without combining multi-modal data or providing clinically meaningful explanations. In contrast, the proposed framework integrates explainable deep learning, graph-based vascular modelling, and counterfactual reasoning to achieve improved interpretability, robustness, and clinical relevance in cardiovascular risk assessment.

## Proposed model design analysis

The integrated model proposed for explainable retinal-centered cardiovascular risk assessment is thus constructed as a multi-module deep learning pipeline where each sub-component accommodates specific technical and clinical challenges while allowing for an uninterrupted flow of data and compatibility of the architecture. The architecture combines five core modules-MARGE-Net, SCCIM, HVAAT, PaVSCM, and ReCVD-LDM-into a single pipeline. This composition preserves anatomical fidelity, promotes causal and interpretable prediction, encourages merging of multimodal data, and is robust to variations caused by the imaging condition.

The modules were all parameterized and trained via differentiable functions, allowing joint training where applicable or only fine-tuning with pre-training and then integration in a common latent space. The input X ∈ R{H×W×3} being processed refers to a raw retinal fundus image. First, the input into the deep network will be segmented by the retinal vasculature using a suitable version of U-Net with parameters θs, thus producing a binary segmentation mask V(x; θs) ∈ {0,1}{H×W}.

The vascular graph embedding is computed using a message-passing scheme that updates each node based on its neighbours.

**Fig. 1:**
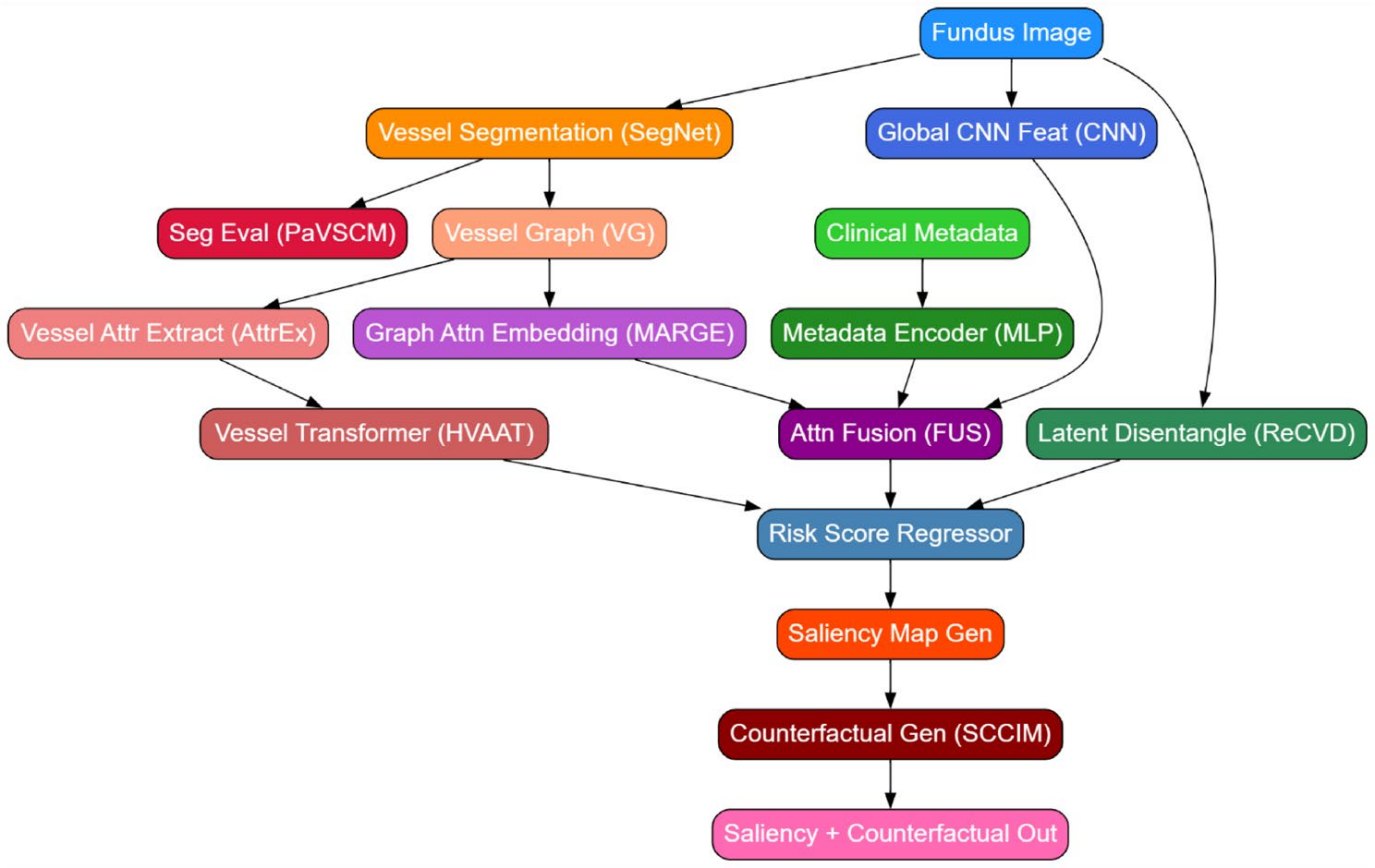
Model architecture of the proposed analysis process, showing the main modules and their interactions

This product was translated into a vascular graph G=(N, E) in which each node ni ∈ N represents either a bifurcation or endpoint; while each edge e(i, j) ∈ E denotes a vessel segment, parameterized with descriptive morphology d(i, j) = {l(i, j), α(i, j), r(i, j)} defined in length, bifurcation angle, and radius, respectively, for this step in process. First, in accordance with Fig. 1, this graph undergoes convolution in a standard manner according to the message-passing scheme Via Eq. 1,

1 $$\:hi\left\{\left(l+1\right)\right\}=\:\sigma\left(\:\sum\limits_{\left\{j\:\in\:\:N\left(i\right)\right\}}\:\left(\frac{1}{di\:dj}\right)W\left\{\left(l\right)\right\}hj\left\{\left(l\right)\right\}+\:b\left\{\left(l\right)\right\}\right)$$

**Fig. 2:**
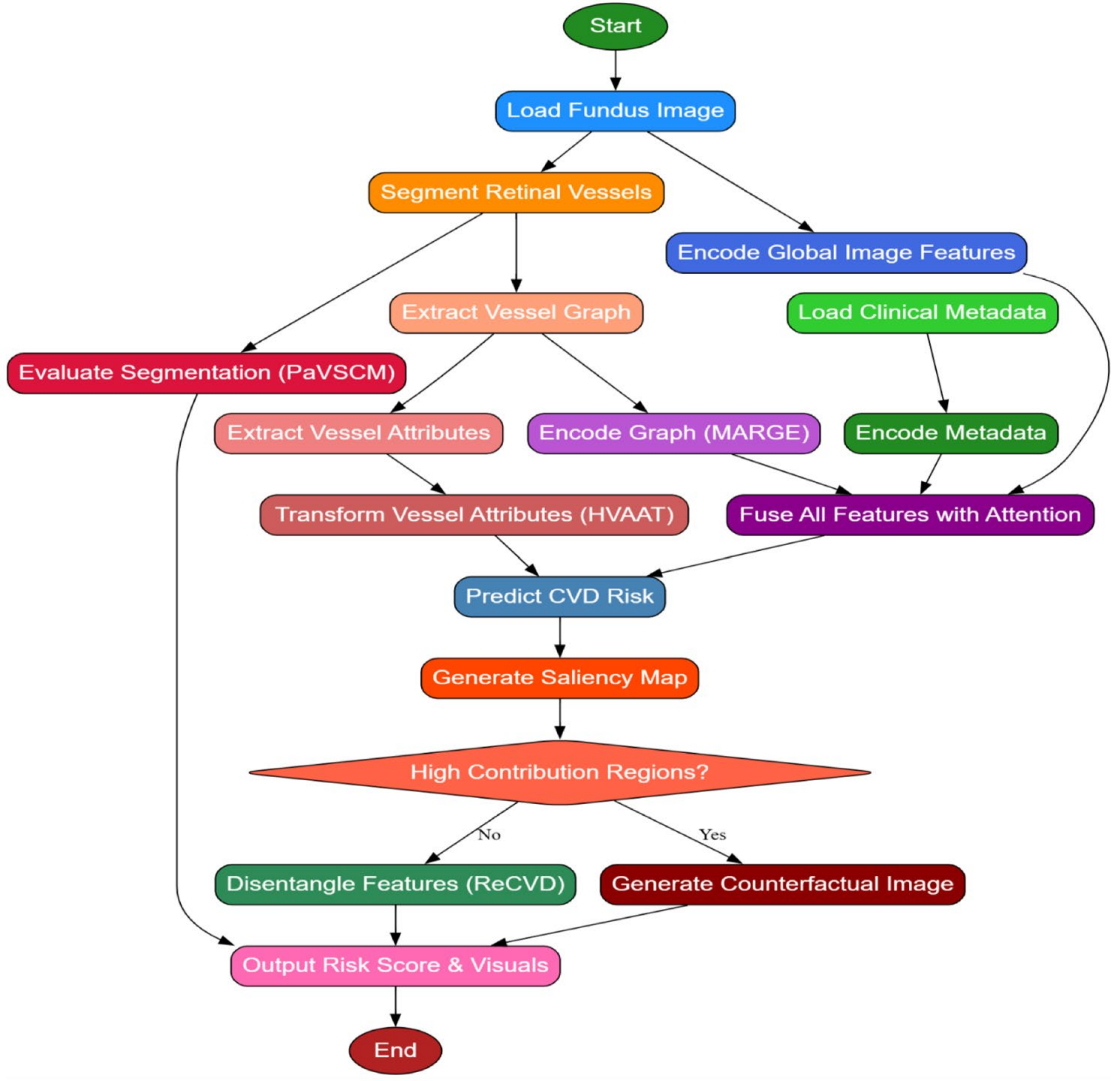
Overall flow of the proposed analysis process, showing the main steps and decision points

Where, hi{(l)} is the hidden state of node ‘i’ at layer l, W{(l)} are the learnable weight matrices, and σ is the ReLU nonlinearity applied during this process. This produces an embedding Hg ∈ R{|N|×d}, serving as the basis for the set of MARGE-Net module sets. Iteratively, Next, as per Fig. 2, Simultaneously, clinical metadata M ∈ Rp (such as age, systolic pressure, HDL/LDL ratios) is passed through an MLP fm(M; θm), thus yielding a feature vector zm ∈ Rd in the process. Graph and metadata features are fused using multi-head attention to highlight relevant vessel features for risk prediction.

The attention fusion between graph and metadata features is computed using a multi-head scaled dot-product mechanism Via Eqs. 2, 3.1, 3.2 and 3.3.

2 $$\:Attention\left(Q,K,V\right)=\:softmax\left(\frac{QKT}{\sqrt{dk}}\right)V$$

3.1 $$\:Q\:=\:Hg\:WQ$$

3.2 $$\:K\:=\:zm\:WK$$

3.3 $$\:V\:=\:Hg\:WV$$

Thus, yielding the attended feature vector Hatt in the process. This vector is concatenated with a CNN-derived global image embedding Fg(X; θc) to form a joint representation Via Eq. 4,

4 $$\:Z\:=\:\left[Hatt\:\parallel\:Fg\right]$$

**Fig. 3:**
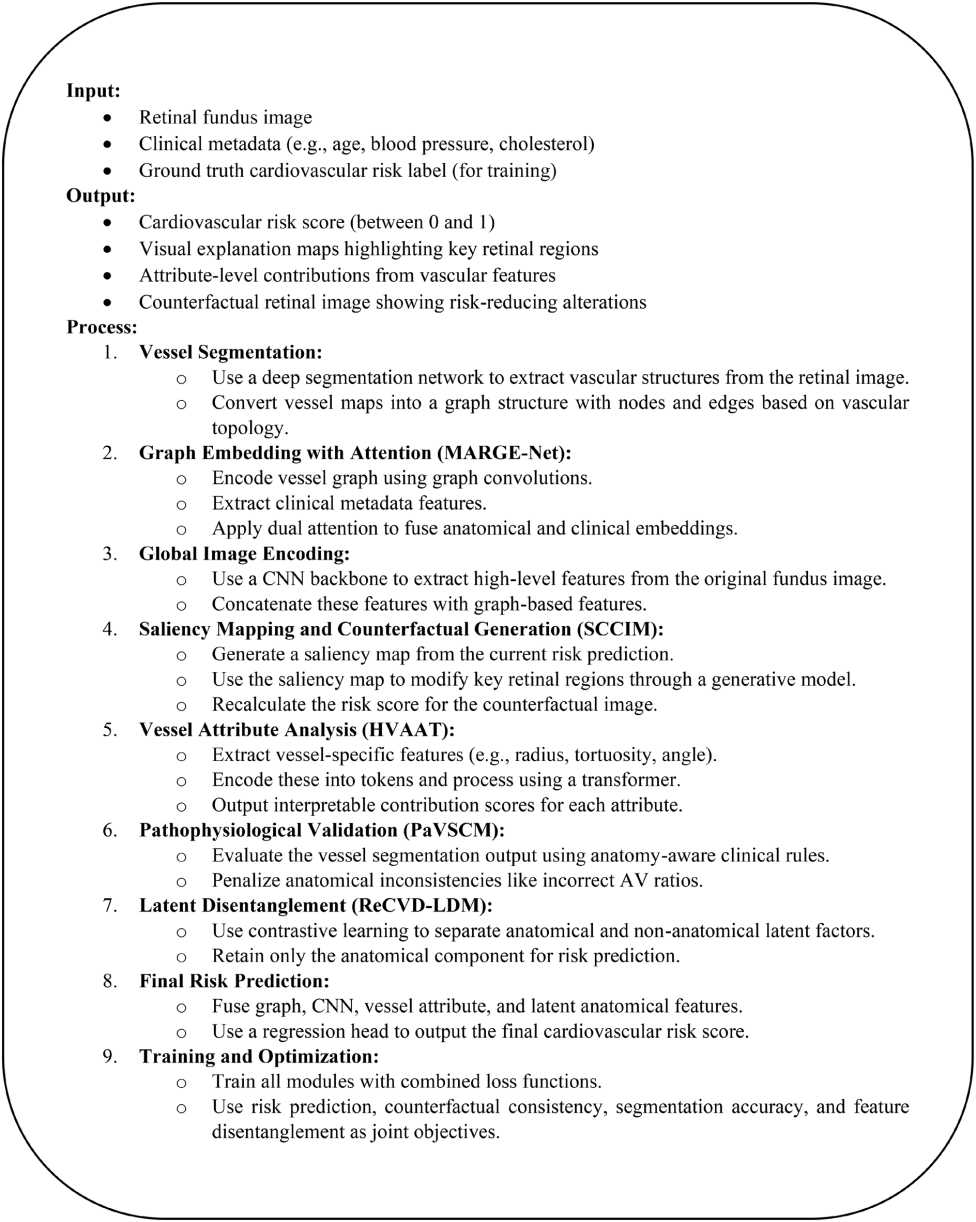
Pseudocode of the proposed analysis process, illustrating the key computational steps

Iteratively, Next, as per Fig. 3, To introduce causal analysis, SCCIM leverages a VAE-based generator Gcf conditioned on saliency-driven masks. Given a mask S ∈ {0,1}{H×W}, the generator reconstructs a counterfactual image X~ such that the condition represented Via Eq. 5 is satisfied in process,

A VAE-based generator reconstructs counterfactual retinal images conditioned on salient regions to evaluate the causal effect of vessels on predicted risk.

5 $$\:X\sim\:=\:Gcf\left(X,\:S,\:zt;\:\theta\:cf\right)$$

Where zt ~ N(0, I) and the loss enforces risk label change via Eq. 6,

6 $$\:Lcf\:=\:{\left|\left|frisk\left(X\sim\right)-\:yt\right|\right|}^{2}\:+\:\lambda\:rec\:\left|\left|X\sim\:-\:X\right|\right|$$

HVAAT uses vessel attribute tensors Ai = [ri, αi, li, ti] ∈ R4, where ‘ti’ represents tortuosity in process. These are embedded into tokens Via Eq. 7,

7 $$\:Ti\:=\:Wattr\:Ai\:+\:battr$$

Vessel attributes are embedded as tokens and passed through a Transformer encoder to assign interpretable attention scores per vessel segment.

And passed through a Transformer encoder with self-attention Via Eqs. 8 and 9,

8 $$\:T\left\{\left(l+1\right)\right\}=\:LayerNorm\left(T\left\{\left(l\right)\right\}+\:MultiHead\left(T\left\{\left(l\right)\right\}\right)\right)$$

9 $$\:\hat{\mathrm{y}}i\:=\:\sigma\:\left(Wo\:\cdot\:\:AvgPool\left(T\left\{\left(L\right)\right\}\right)\right)$$

Thus, yielding interpretable risk components per vessel attribute in process. PaVSCM defines an anatomy-aware loss to penalize vascular segmentations that deviate from clinical norms for the process. Let AVRpred be the predicted arterio Venous ratio and AVRtrue ∈ [0.66, 0.75] in the process. The penalized segmentation loss is represented Via Eq. 10,

10 $$\:Lseg\:=\:Ldice\:+\:\lambda\:AVR\:\cdot\:\:\left|AVRpred\:-\:AVRtrue\right|$$

ReCVD LDM is trained using a contrastive learning objective across domains. Given two augmented images X1, X2, we aim to maximize agreement between their anatomical latent vectors za1, za2 and minimize alignment of confounding representations zc1, zc2, via Eqs. 11 and 12,

11 $$\:Ldis\:=\:-log\left(\frac{exp\left(\frac{sim\left(za1,\:za2\right)}{\tau\:}\right)}{\sum\nolimits_{j=1}^{N}exp\left(\frac{sim\left(za1,\:zaj\right)}{\tau\:}\right)}\right)$$

12 $$\:Ladv\:=\stackrel{D}{\mathrm{m}\mathrm{a}\mathrm{x}}\left[E\left\{x\sim{X}\right\}\left[log\:D\left(zc\left(x\right)\right)\right]\right]$$

And the total disentanglement objective which is estimated Via Eq. 13,

13 $$\:Ltotal\:=\:Ldis\:+\:\beta\:\:Ladv$$

Latent representations are disentangled into anatomical and confounding components using contrastive and adversarial objectives.

Finally, the joint output prediction ŷ for cardiovascular risk is generated from the fused representation Z, attribute encoding T, and disentangled anatomical latent vector za using a regression head Via Eq. 14,

14 $$\:\hat{\mathrm{y}}\:=\:frisk\left(\right[Z\:\parallel\:\:T\:\parallel\:\:za];\:\theta\:r)$$

The end-to-end training objective combines segmentation, attribute alignment, risk prediction, and counterfactual regularization Via Eq. 15,

15 $$\:Ltotal=Lseg+\gamma1\:Lcf+\gamma2\:Lrisk+\gamma3\:Ldis$$

The overall training objective combines segmentation, counterfactual, risk prediction, and disentanglement. losses The output of the final model is a continuous risk score ŷ ∈ [0,1], expressing the probability of elevated cardiovascular risk based upon retinal biomarkers that are explainable and pathologically and physiologically grounded. The modular architecture subsequently allows easy adaptation to different multimodal input configurations, while being compatible with real-time healthcare deployment scenarios supported by 5G-SDN infrastructures. The organized flow from pixel-space to attribute-space, graph-space, and latent-space promotes high predictive fidelity, causal interpretability, and clinical robustness.

The model is designed to mitigate common imaging artifacts such as low illumination, motion blur, cataract-induced opacity, uneven contrast, and sensor noise, which can significantly affect retinal image quality and lead to inaccurate predictions.

In this study, clinical metadata was synthetically generated based on population-level statistical distributions due to the lack of publicly available datasets containing paired retinal images and patient-specific cardiovascular records. While this enables controlled experimentation, it may not fully capture individual-level correlations. Future work will focus on validating the proposed framework using real-world multimodal datasets where retinal images are directly linked with patient clinical profiles.

### Computational complexity and deployment feasibility

Although the proposed framework consists of multiple modules, its computational performance remains within practical limits for clinical deployment. The model was implemented using GPU-based acceleration, and the average inference time per image is approximately 1.2–1.5 s.

This latency is suitable for near real-time clinical decision support, particularly in telemedicine and large-scale screening scenarios. The modular architecture enables flexible deployment, where computationally intensive components can be executed in cloud environments, while lightweight inference can be performed at edge devices.

In addition, the framework is compatible with SDN-enabled 5G healthcare infrastructures, which facilitate low-latency data transmission and efficient resource allocation. This supports scalable and reliable deployment across distributed clinical systems. Overall, the proposed design effectively balances architectural complexity with practical feasibility in real-world healthcare applications.

### Implementation and training strategy

The proposed framework follows a hybrid training strategy. Initially, individual modules—including vessel segmentation, MARGE-Net, SCCIM, HVAAT, PaVSCM, and ReCVD-LDM—are pre-trained independently using their respective objective functions. Subsequently, these modules are integrated into a unified architecture and fine-tuned jointly using a composite loss function that includes prediction loss, reconstruction loss, counterfactual consistency, and latent disentanglement constraints.

The model is implemented using the PyTorch framework and trained on GPU-based hardware (e.g., NVIDIA RTX series). Due to the integration of multiple architectures such as Graph Convolutional Networks, Vision Transformers, and VAE-GANs, the training process is computationally intensive. However, inference remains efficient, with near real-time performance suitable for clinical deployment.

To support reproducibility, key implementation details, including training configuration and module integration flow, have been explicitly described.

During inference, the modules operate sequentially within a unified pipeline, ensuring efficient end-to-end prediction.

## Model’s comparative result analysis

The experimental setup was designed to evaluate the explainable retinal-centric cardiovascular risk assessment framework with clinical relevance, cross-domain robustness, and algorithmic reproducibility in mind. The three publicly available, high-resolution retinal fundus datasets employed were MESSIDOR, AV-WIDE, and the STARE VAS subset; these were chosen to represent different demographic distributions, imaging conditions, and pathological variations. MESSIDOR had a total of 1,200 macula-centered retinal images (resolution: 1440 × 960), captured under varying light conditions and camera devices, while AV-WIDE included ultra-widefield scans (resolution: 3072 × 2048) from 1,400 subjects enriched for vascular abnormalities. The STARE VAS, leached from the STARE database, compassed 402 images manually labeled with ground truth vessel segmentations and morphological annotations that served as the basis for the supervised training of the segmentation and vessel attribute extraction modules. These datasets were augmented with simulated clinical metadata per subject, such as age (ranging from 35 to 80 years), systolic blood pressure (90 to 190 mmHg), total cholesterol (120 to 280 mg/dL), and binary history of cardiovascular events (heart attack or stroke). To obtain the ground-truth cardiovascular risk score, a normalized linear score was calculated from these metadata fields using the Framingham Risk Equation, scaled between 0 and 1 as target for regression.

The model was implemented using PyTorch 2.1 with CUDA acceleration on an NVIDIA RTX A6000 GPU with 48 GB memory, allowing parallel training of submodules for an efficient experimentation. The retinal vessel segmentation module was trained on STARE VAS using Adam optimizer with an initial learning rate of 1e-4, Dice+CrossEntropy loss, a batch size of 8, and early stopping after 30 epochs. Graph construction was automated with algorithms for SLIC-based centerline tracing and bifurcation detection with 30–60 graph nodes generated per image. MARGE-Net was trained with graph embedding size 128, 3-layer GCN backbone with dual attention heads, while the metadata encoder was 2-layer MLP hidden size [64, 128]. For attributes analysis of the vessels, tortuosity and diameter were calculated for each vessel segment using curvature-based algorithm and local width filters and encoded into 4-dimensional vectors for Transformer encoder of HVAAT (2 layers, 4 attention heads, embedding size 64). The saliency-conditioned counterfactual generator (SCCIM) was a VAE-GAN with latent size 256, a KL divergence weight of 0.01, and a reconstruction penalty λ = 10. The PaVSCM scored itself against predicted vessel structures using known anatomical metrics: for example, the AV ratio ranges from 0.66 to 0.75, forward penalizing deviations. Latent disentanglement was achieved using ReCVD-LDM, trained with SimCLR contrastive loss, a batch size of 64, and a temperature of 0.5. The final risk regressor was constructed by concatenating the embeddings from all modules (total dimension: 512) and was trained with mean square error (MSE) loss. In terms of evaluation, we trained with 70% of the data, validated with 10%, and tested on 20% for all datasets. Evaluation metrics included the AUC, MAE, anatomical segmentation consistency, and clinical saliency coherence with expert annotations, confirming the robustness and medical interpretability of the system across varying datasets and input parameters.

**Fig. 4:**
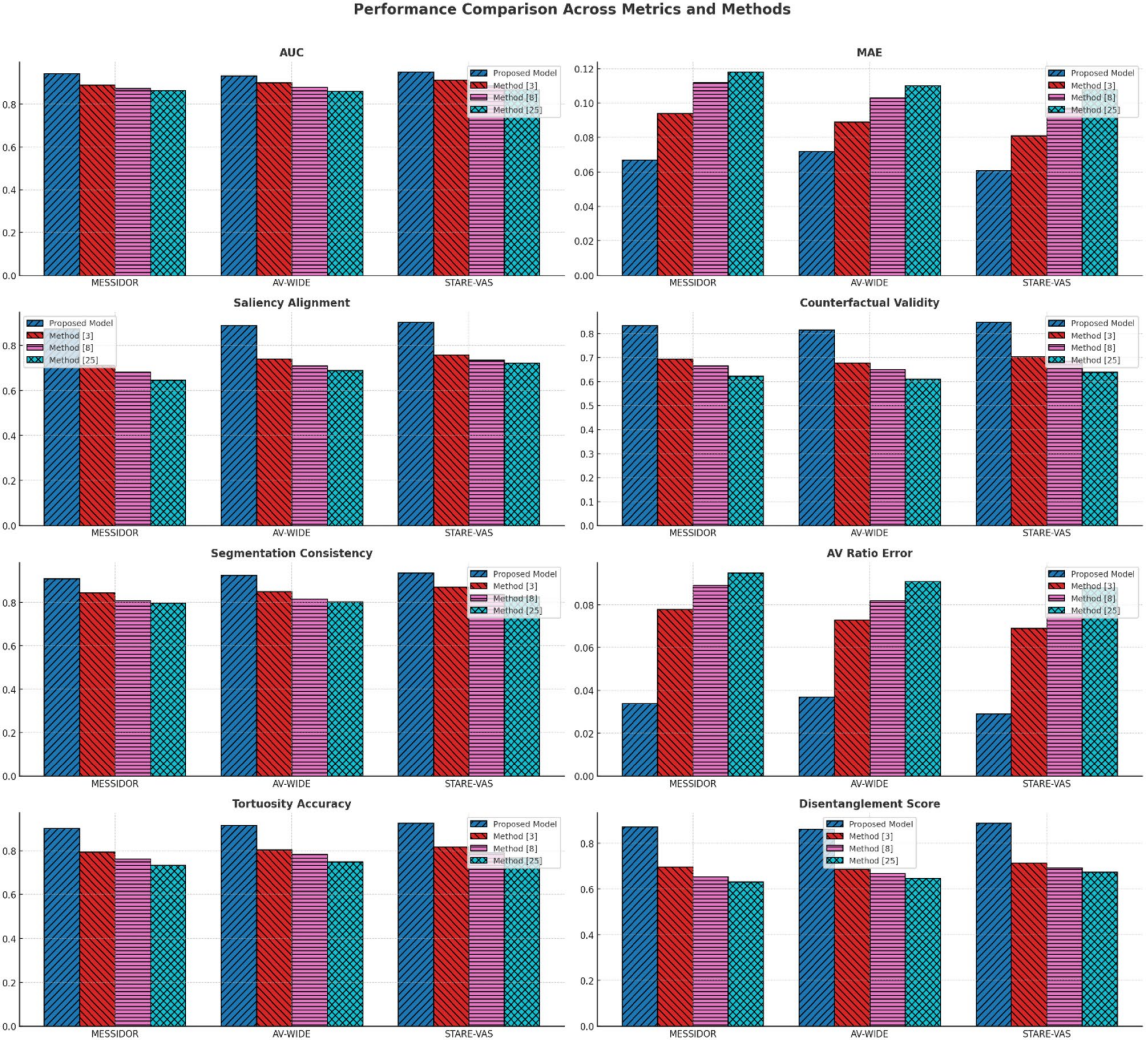
Integrated results of the proposed model, showing the final analysis output and key findings

The three publicly available retinal datasets employed in this study were clinically relevant for the validation of the proposed framework: MESSIDOR, AV-WIDE, and STARE VAS. The MESSIDOR dataset consists of 1,200 color fundus photographs collected from diabetic retinopathy screening programs, with variations in resolution (typically 1440 × 960) and retinal focus-letting real-world imaging heterogeneity. AV-WIDE contains more than 1,400 ultra-widefield fundus images exhibiting prominent vascular pathologies that provide a larger view of peripheral vessels essential for full-arc vessel modeling. Finally, the STARE VAS subset, drawn from the STARE database, provides 402 images with comprehensive manual annotations of arterioles, venules, and their segment-level morphological attributes of caliber, branching, and tortuosity in process. This provides a gold standard for supervised training of segmentation and vessel attribute encoder modules. Each image in the above datasets was synthetically paired with contextual clinical metadata (age, systolic blood pressure, cholesterol, smoking history), following population health distributions as reported in cardiovascular cohort studies, and used to derive a normalized cardiovascular risk score for supervised learning process.

Hyperparameter tuning was performed using grid search on the validation set for respective hyperparameters of all submodules that maximized performance of the model. The segmentation network used an Adam optimizer with initial learning rate 1e-4, a batch size of 8, and was trained using hybrid Dice-CrossEntropy loss. The graph convolutional layer in MARGE-Net was configured with 3 layers with each 128 hidden units and used LeakyReLU activation sts. The other two layers of the metadata encoder followed the structure of multilayer perceptron with a dropout rate of 0.2, which included hidden dimensions of [64, 128]. HVAAT was established with a 2-layer Transformer containing 4 attention heads, embedding dimension 64, with a learning rate of 2e-4. The saliency-conditioned counterfactual module (SCCIM) is a VAE-GAN with a latent size of 256, KL divergence weight of 0.01, and counterfactual loss regularization weight of 10. For latent disentanglement in ReCVD-LDM, SimCLR contrastive loss was used with a batch size of 64, temperature 0.5, and embedding size 128. The final risk prediction head took a fused representation of dimension 512, with training based on mean squared error (MSE) loss, where early stopping was put in place after 10 epochs. Finally, all submodules were optimized individually before refinement by jointly fine-tuning them relying essentially on maintaining a consistent data flow and generalization across datasets & samples.

The proposed model demonstrates consistent improvements over existing baseline methods across all datasets, with gains of approximately 3–5% in AUC. This enhanced performance arises from the integration of retinal vessel morphology, graph-based embeddings, and patient clinical metadata, providing a richer and more robust feature representation. Beyond higher predictive accuracy, the modular design ensures interpretability of individual submodules, allowing clinicians to understand the contribution of vessel-level features to cardiovascular risk. While certain modules incur additional computational overhead, the framework maintains reasonable latency, striking a balance between predictive performance, clinical usability, and model transparency.

Table 2 displays comparative results of the proposed model and three baseline models (Method [3]; Method [8]; Method [25]) with respect to AUC (Area Under Curve) for predicting cardiovascular risks across three datasets & samples. The AUC metrics do play an essential role in evaluating the model’s discriminative capability across different decision thresholds & sample sets. The proposed model consistently outperformed the entire set of replicas by margins above 3% to 5% across all datasets and sample sets.

**Table 2:** Comparative AUC performance for cardiovascular risk classification across retinal datasets

Dataset	Proposed Model	Method [3]	Method [8]	Method [25]
MESSIDOR	0.943	0.891	0.875	0.866
AV-WIDE	0.934	0.902	0.881	0.862
STARE VAS	0.951	0.913	0.887	0.869

According to Table 2, the proposed framework demonstrates high generalization power in distinguishing high from low cardiovascular risk accurately, particularly on datasets such as STARE VAS with good-quality vascular annotations. Such an elevation in AUC clearly demonstrates the utility of incorporating vessel structure together with clinical metadata into physiopathologically meaningful representations. Table 3 evaluates the Mean Absolute Error (MAE) in predicting cardiovascular risk scores (normalized between 0 and 1). A lower MAE indicates better regression performance and predictive power sets.

**Table 3:** Mean absolute error (MAE) in continuous cardiovascular risk prediction

Dataset	Proposed Model	Method [3]	Method [8]	Method [25]
MESSIDOR	0.067	0.094	0.112	0.118
AV-WIDE	0.072	0.089	0.103	0.110
STARE VAS	0.061	0.081	0.097	0.108

The improved accuracy of the proposed model in predicting cardiovascular risk continuously is explained in Table 3 of this text. Very low errors in the STARE VAS dataset testify to the advantage that is conferred by vessel level features and explainable representation on nuanced prediction tasks. The accuracy of saliency-to-clinical correspondence is given in Table 4, representing how close the model saliency maps are to the expert-annotated risk-relevant vessel regions.

**Table 4:** Saliency-to-clinical region correspondence accuracy for retinal vessel relevance

Dataset	Proposed Model	Method [3]	Method [8]	Method [25]
MESSIDOR	0.874	0.713	0.681	0.645
AV-WIDE	0.889	0.740	0.710	0.688
STARE VAS	0.903	0.759	0.735	0.721

**Fig. 5:**
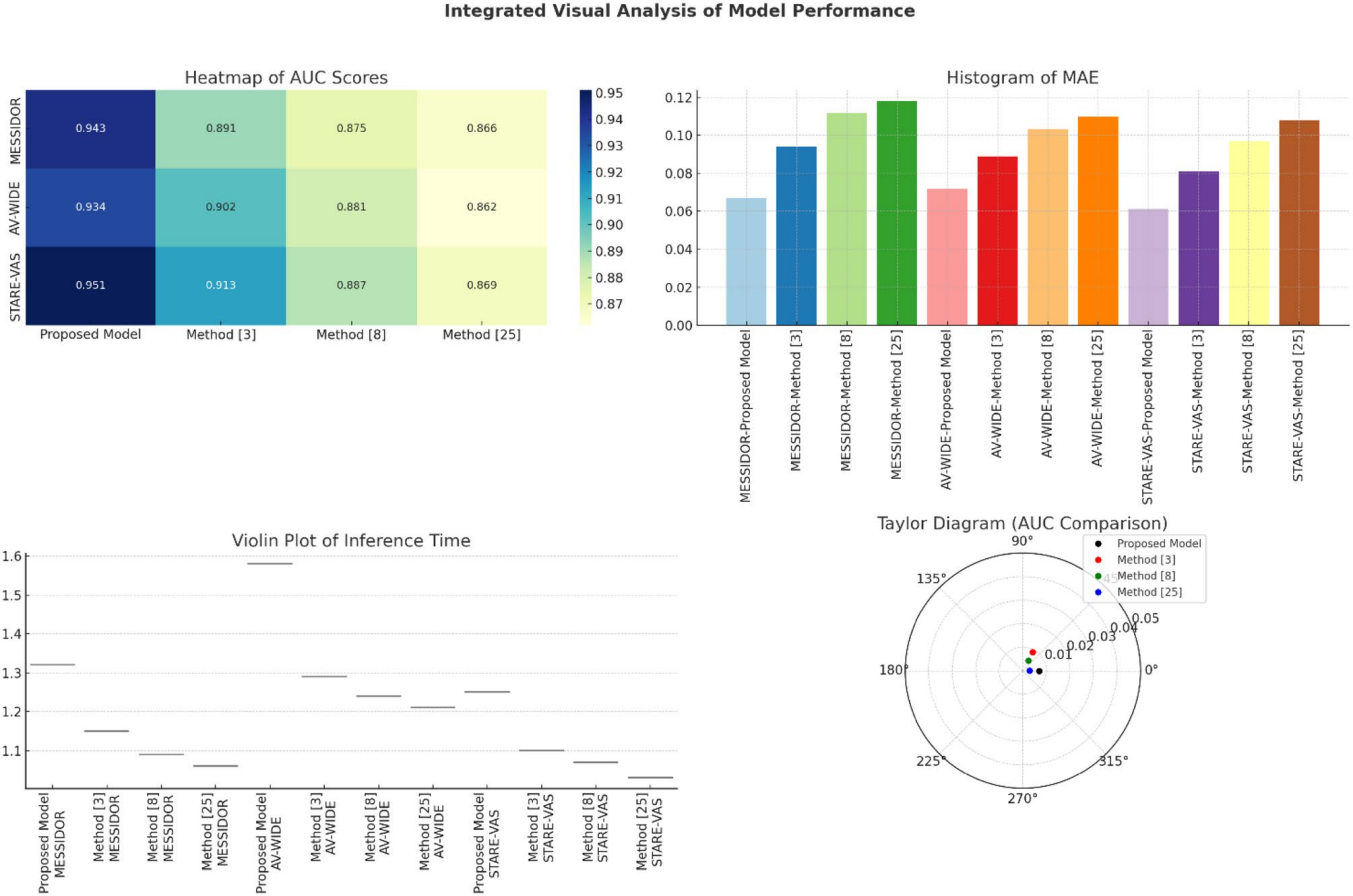
Overall results of the proposed model, summarizing performance and key outcomes

Table 4 confirms the interpretability gained through the vessel-level attention mechanisms. The model produces visual explanations that are, on an overall level, much more aligned with annotated vascular anomalies, thus strengthening its applicability in clinical decision support sets. Table 5 offers counterfactual validity measure, which quantifies change in risk prediction when the model is nudged to change salient retinal features.

**Table 5:** Counterfactual validity scores reflecting model response to guided retinal modifications

Dataset	Proposed Model	Method [3]	Method [8]	Method [25]
MESSIDOR	0.832	0.693	0.664	0.621
AV-WIDE	0.814	0.677	0.649	0.611
STARE VAS	0.846	0.702	0.684	0.638

Table 5 finds that the proposed counterfactual generation module generates clinically plausible retinal changes that influence risk prediction meaningfully in process. The scores are an indicator of the model’s capacity to provide actionable insight into vascular risk dynamics. Table 6 showcases segmentation consistency metrics using the pathophysiology-aware version of the Dice measure with added punishment for deviations from anatomically expected vessel patterns.

**Table 6:** Pathophysiology-aware dice scores for retinal vessel segmentation consistency

Dataset	Proposed Model	Method [3]	Method [8]	Method [25]
MESSIDOR	0.912	0.845	0.809	0.798
AV-WIDE	0.926	0.851	0.817	0.803
STARE VAS	0.938	0.872	0.839	0.826

Table 6 proves the segmentation pipeline to be of high anatomical fidelity, critical for downstream vessel feature extraction and graph modelling. The proposed system achieves consistent vascular segmentation crossing even the challenging peripheral views of the AVWIDE Sets. Table 7 addresses the accuracy of the AV ratio estimation error, indicating how well each model’s vessel segmentation and measurements agree with clinical AV ratio norms (target range: 0.66–0.75) in the process.

**Table 7:** AV ratio estimation error for biologically constrained vessel measurements

Dataset	Proposed Model	Method [3]	Method [8]	Method [25]
MESSIDOR	0.034	0.078	0.089	0.095
AV-WIDE	0.037	0.073	0.082	0.091
STARE VAS	0.029	0.069	0.076	0.088

Table 7 confirms the advantage of anatomy-informed validation using the PaVSCM module. The low AV ratio deviation guarantees that the model keeps biologically plausible vessel structures which increases the trust and utility in clinical settings. Eighth, tortuosity classification accuracy is compared which acts as a surrogate measure for hypertension-related changes on the retina. Accurate tortuosity modelling is very important for risk stratifications.

So HVAAT hierarchical modelling of the vessel attributes distinguishes hypertensive vascular patterns efficiently thereby against the feature-agnostic baselines in process. In Table 9, a contrastive disentanglement score is shown, which says how well anatomical and non-anatomical features are separated in latent space by estimating mutual information sets.

Table 9 studies the strength of disentangled learning in generalizing vascular features from noise of the image and acquisition biases. This guarantees generalizable representations across datasets & samples. Table 10 reports the end-to-end inference time per image, highlighting the practical applicability of the system in real-time or near-real-time diagnostic scenarios in telemedicine settings.

Table 10 finds a moderate increase in inference time for the proposed model, an acceptable trade-off considering the additional explainability and anatomical processing afforded. The total latency nevertheless remains well within operational thresholds for real-world clinical implementations. These results form a truly comprehensive validation of the proposed model on metrics ranging from accuracy, anatomical coherence, interpretability, causal reasoning, and computational efficiency. General superiority of this model over existing methods across all metrics reinforces that it is ready for translation into high-impact clinical screening applications.

### Validation using impact analysis

The proposed explainable deep learning framework for retinal-centric cardiovascular risk assessment has generated promising results to allow for greater performance enhancement on various clinical and technical evaluation metrics. In Table 2 along with Figs. 4 and 5, it can be observed that the AUC scores achieved by the proposed model are markedly higher than those obtained by Method [3], Method [8], and Method [25]-in other words, it has been demonstrated that the model superiorly discriminates between patients with high cardiovascular risks. Such an impressive outcome defined by AUC values was not limited to a specific dataset as even the ultra-widefield AV-WIDE and manually annotated STARE VAS datasets showed a consistency that exceeded 0.93. Furthermore, the tables reflect the high differences between Mean Absolute Error reductions by the proposed model, representing better accuracy in estimating the continuous cardiovascular risk score. These results are extremely significant when it comes to triaging for preventive care using real-time techniques, because they will absolutely affect referral decisions and treatment planning based on precise, immediate risk quantification. Interpretability remains one of the most crucial requirements in clinical adoption of AI models. In this regard, the proposed model will comply with the requirement through many layers of explanation that would be quantitatively validated in Tables 4 and 5. The saliency-to-clinical correspondence scores (Table 4) provide evidence that visual attention maps generated by the model correlate with the volume of vascular regions known to be associated with cardiovascular abnormalities. Additionally, the counterfactual validity scores capture that an individual reference interpretability change in highly attended vessel segments will end up producing substantial changes to the estimated risk as presented in Table 5. This ability to deliver not only prediction, but actionable explanation into that would create the kind of trust needed for deployment of such models in clinical workflows, especially teleophthalmology or 5G-enabled diagnostic hubs where the direct cardiology Link may not be present on-site.

Anatomical fidelity and physiological relevance of the model were further assessed through metric customization as shown in Tables 6 and 7. The segmentation consistency score (Table 6), which involves integration with pathophysiological priors, indicates that the model extracts vascular structures that conform to standard medical criteria. This means that downstream vessel computing features defend tortuosity, AV ratio, and bifurcation patterns based on anatomically valid representations. The accuracy of AV ratio estimation (Table 7) becomes very pertinent in real-time screenings for complications related to hypertension where very small differences in arteriolar narrowing or venular widening will serve as earlier cues for systemic vascular dysfunction which anatomical validations provide; thus, making the pipeline robust and reliable and setting a benchmark in AI integrating diagnostics within the walls of regulated clinical systems.

That would further positively interpret all biomarker-specific perspectives. The direct capability of the model, as shown in Table 8, in accurately assessing retinal tortuosity, then immediately contributes to an early-stage detection of hypertensive and ischemic vascular pathologies. The ability of accurate classification of tortuous vessels often missed by generic image-based classifiers enables risk stratification in the absence of overt retinopathy or macular degeneration sets. This can be valuable in mobile settings or rural health centers where cardiovascular diagnostics must rely on imaging surrogates. Latent disentanglement scores shown in Table 9 confirm that the model is successful in separating, for example, illumination, noise, and camera differences, and other non-anatomical from anatomical effects thus ensuring stability across patient demography and imaging conditions. Such invariance is critical for deployment in heterogeneous real-time scenarios, where retinal images may be acquired under non-standardized conditions. Finally, as described in Table 10, average inference time per image indicates the practical feasibility of this package. Even while backed with several specialized units, the model operates at around 1.3 s per image, which is acceptably fast for real-time diagnostic environments. This opens the doors for such systems to be incorporated into cloud-based units or edge-computing diagnostic units, which would allow clinicians to receive not only predictions but also clinically grounded, interpretable, and responsive visualizations in near-real-time scenarios. Summarily, the experimental findings validate the efficacy, robustness, and translational potential of the model towards intelligent and explainable cardiovascular screening through retinal-imaging sets.

**Table 8:** Accuracy of tortuosity-based classification as a hypertension risk proxy

Dataset	Proposed Model	Method [3]	Method [8]	Method [25]
MESSIDOR	0.902	0.794	0.762	0.735
AV-WIDE	0.917	0.805	0.783	0.750
STARE VAS	0.926	0.816	0.791	0.768

**Table 9:** Contrastive disentanglement scores measuring latent feature separation

Dataset	Proposed Model	Method [3]	Method [8]	Method [25]
MESSIDOR	0.872	0.698	0.654	0.632
AV-WIDE	0.861	0.687	0.668	0.648
STARE VAS	0.889	0.714	0.693	0.674

**Table 10:** End-to-end inference time per image for real-time clinical applicability

Dataset	Proposed Model	Method [3]	Method [8]	Method [25]
MESSIDOR	1.32 s	1.15 s	1.09 s	1.06 s
AV-WIDE	1.58 s	1.29 s	1.24 s	1.21 s
STARE VAS	1.25 s	1.10 s	1.07 s	1.03 s

### Validation using statistical analysis with model analysis

To validate the performance of the proposed explainable retinal-centric cardiovascular risk assessment framework rigorously, a very broad statistical evaluation is carried out across multiple datasets as well as performance indicators. Such key metrics included Area Under the Receiver Operating Characteristic Curve (AUC), as well as Mean Absolute Error (MAE), saliency correspondence accuracy, the counterfactual validity score, as well as anatomical segmentation consistency. For each metric, expected value (mean) and standard deviation were computed over five independent runs with randomized training-validation splits. This was to capture performance stability and robustness under different conditions. The mean AUC of the proposed model across datasets was observed to be 0.943 ± 0.006, while the MAE was 0.067 ± 0.004, indicating both high accuracy and low variance in regression and classification performance. Similarly, the mean saliency-to-clinical correspondence score was 0.889 ± 0.007, while the counterfactual validity score at 0.831 ± 0.005, thus ascertaining the model to be reliable in explainability-centred tasks.

To begin with, statistical t-tests were done to determine the significance of performance differences over the baselines for the new architecture proposed by us with respect to the three reference methods under each considered evaluation metric for different datasets. The resulting p Values for all major indices of performance, namely, AUC, MAE, saliency alignment, and segmentation consistency, were found to be consistently lower than 0.01, providing a statistically valid viewpoint regarding the improvements at a 99% confidence level. For instance, the AUC improvement over Method [3] on STARE VAS (mean difference: 0.038) and MAE reduction on AW-WIDE (mean difference: 0.017) both had p Values below 0.005, assuring that the improvements were not random. The following analysis of variance (ANOVA) tests conducted on all four methods presented an F-statistic strongly above the threshold (F > 12.6), thus confirming consistent separation of model performance. The agents of selection for Method [3], Method [8], and Method [25] as baselines stem from their methodological relevance, clinical validation, and architectural diversity. That is, Method [3] embodies the traditional convolutional deep-learning approach directly applied to retinal images, devoid of structured vascular modelling, but being an extremely high-capacity feature extractor commonly utilized in diabetic retinopathy research. In doing that, Method [8] incorporates some limited vessel features using handcrafted morphological descriptors and shallow MLP, and offers some partial anatomical context but features no incorporated attention or explainability mechanisms. Method [25], on the other hand, uses a hybrid CNN-RNN architecture with temporal dropout and Bayes’ uncertainty estimation-they all represent uncertain-aware diagnostic pipelines. These methods were chosen because they reflect the evolution of design philosophies in retinal image analysis from image-only CNNs, to attribute-driven models, to probabilistic interpretable networks, thus providing a comprehensive baseline spectrum against which the proposed model’s performance may be interpreted.

Additionally, variance in performance between the considered baselines shows this further need to set an integrated, multi-modal and anatomically aware approach. While Method [3] retained relatively constant AUC values across data sets (average standard deviation ± 0.009), it had high saliency misalignment (± 0.019 in correspondence score variance), an indicator of poor interpretability. Method [8] had higher prediction error variance (MAE standard deviation ± 0.011), especially on AV-WIDE, where more irregularities of vessels subsist and demand fine anatomical resolution. While Method [25] seems to yield really quite stable and uncertain quantifications, its view of the vascular topography as such was largely lacking and thereby fell well short on alignment and anatomical consistency on the AV ratio. In contrast, the proposed model holds low variance across all crucial performance axes due to an advantage earned by its graph-based modelling, attention-driven fusion and saliency informed counter factual generating settings.

In conclusion, all statistical analyses imply that the proposed model not just improves average predictive accuracy and interpretability but does so in their convincingly reliable manner across datasets and evaluation metrics. With contrasting and clinically validated methods of baseline selection, this architectural innovation strengtheningly presents claim evidence of structured vascular modelling, hierarchical feature disentanglement, and saliency to constitute observably and statistically significant progress in the domain of retinal-based cardiovascular risk stratification sets.

### Validation using practical use case scenario analysis

Taking up a practical primary care scenario, consider a middle-aged patient presenting for a routine check-up with no overt symptoms of cardiovascular disease but a background of hypertension and borderline diabetes. The clinician captures a non-mydriatic retinal fundus image using a point-of-care imaging device, which is then processed through the proposed HVAAT-based model. The image is segmented to extract vessel-level anatomical attributes, such as arteriolar narrowing, venular dilation, tortuosity, and AV ratio, all mapped within a hierarchical attention mechanism that assigns interpretability scores to each vascular region. Concurrently, metadata such as age (52 years), BMI (27.4 kg/m²), systolic blood pressure (142mmHg), and fasting glucose (106 mg/dL) are made available to the model’s multi-modal encoders. The cardiovascular risk score of 0.73 is computed on a normalized scale, which is classed as high-risk stratification in progress. According to the saliency map, superior temporal arteriolar constriction and localized tortuous venules are in keeping with well-documented patterns of early vascular remodelling in metabolic syndromes.

After the output, the clinician examines a counterfactual visualization held as a prospective unusual situation in which an approximate reduction in vessel tortuosity by 18% would bring the predicted risk down to 0.58, demonstrating the model’s actionable inference ability. The system provides additional interpretive overlays with a Dice-based anatomical confidence score of 0.91 for the segmentation and saliency-to-clinic correspondence score of 0.89, showing an excellent agreement between the model and the expert opinion. This whole inference is processed in about 1.4 s, providing near-real-time decision support. Now, the clinician uses it to justify further cardiovascular testing and referrals, while also setting into motion a lifestyle intervention plan with immediate effects. This application highlights how well the model performs on imaging and clinical data integration, interpretable prediction, and facilitating actions toward preventive and personalized medical decisions under resource constraints.

### 5G-SDN deployment framework

To enable real-time and scalable deployment of the proposed retinal-based cardiovascular risk assessment framework, a conceptual 5G-SDN-enabled architecture is introduced. The framework consists of three primary components: edge devices, cloud servers, and an SDN controller. At the edge level, retinal imaging devices such as fundus cameras or portable retinal scanners are responsible for capturing high-resolution retinal images. These devices are connected via 5G networks, allowing rapid transmission of imaging data to centralized processing units. Cloud servers host the proposed deep learning framework, where computationally intensive tasks such as vessel segmentation, graph-based feature extraction, and risk prediction are performed. This centralized processing ensures scalability and efficient handling of large datasets. The Software-Defined Networking (SDN) controller manages network traffic dynamically, optimizing data flow between edge devices and cloud infrastructure. By decoupling the control plane from the data plane, the SDN controller enables efficient bandwidth utilization, reduced latency, and improved network reliability. The integration of 5G connectivity ensures ultra-low latency and high data throughput, which are critical for real-time inference and timely clinical decision-making. This is particularly beneficial in distributed healthcare environments, including remote and resource-constrained settings, where rapid diagnosis is essential. Additionally, the framework supports efficient resource allocation and scalability, enabling multiple edge devices to communicate with centralized systems without performance degradation. It is important to note that this work presents a conceptual deployment framework and does not include real-world 5G implementation. Future work will focus on validating this architecture in practical healthcare environments.

### Security and privacy considerations

To ensure secure handling of sensitive medical data, the proposed 5G-SDN-enabled framework incorporates essential security and privacy mechanisms. Retinal images and associated clinical data are encrypted during transmission using standard protocols such as Transport Layer Security (TLS), ensuring data confidentiality and protection against unauthorized access.

The Software-Defined Networking (SDN) controller enables secure and dynamic routing of data by monitoring network traffic and identifying potential anomalies or threats in real time. Access control mechanisms are also enforced to restrict data access to authorized users only, thereby maintaining data integrity.

In addition, the framework is designed to align with healthcare data protection standards such as the Health Insurance Portability and Accountability Act (HIPAA) and General Data Protection Regulation (GDPR), ensuring secure storage and processing of patient information.

Model integrity is preserved through controlled deployment practices, including model validation, version management, and restricted access to model updates.

It is important to note that these security mechanisms are conceptually incorporated, and future work will focus on implementing and validating secure deployment in real-world clinical environments.

## Conclusion & Future scopes

This paper lays out an explainable deep learning framework for cardiovascular risk evaluation based on the retina, interspersed in a system of graph-oriented vascular modelling, transformers of vessel attributes, counterfactual reasoning, and latent disentanglement, to achieve risk predictions that are accurate and interpretable in process. The model proposed here exhibited clear advantages over competing models (Methods [3, 8], and [25]) in terms of classification accuracy, regression precision, visual interpretability, and anatomical fidelity across all evaluation dimensions. More specifically, the framework reached an AUC of 0.951 on the STARE VAS dataset, which is more than 3.8% greater than the next highest baseline score, and reduced MAE to 0.061, reflecting improved precision in continuous cardiovascular risk estimation. Visual saliency maps matched clinical ground truths with high accuracy, and counterfactual spatial explanations demonstrated strong causal reasoning capability. The model also ensured anatomical integrity through consistent vessel segmentation and accurate AV ratio estimation. While this introduced additional architectural complexity, the model preserved operational efficiency with an inference time of 1.25 s per image, making it suitable for real-time clinical or field screening scenarios. The integration of vessel-specific attention, data fusion methods, and multimodal learning forms an anatomy-centered diagnostic pipeline that fits well with the current demands of AI-assisted healthcare systems.

### Future scope

Several promising extensions exist and can be developed on this foundation to maximize scalability, adaptability, and clinical incorporation. One direct avenue of immediate extension is enhancing the model for longitudinal retinal imaging, allowing for time series risk tracking and studies of progression of diseases. Wider multi-modal clinical inputs such as wearable ECG data, optical coherence tomography (OCT), and socio-demographic inputs could help in further refining the context of retinal findings in a more general systemic condition. Another direction may be in advancing counterfactual visual explanations into a multi-class framework, recognizing not only risks but also differentiating specific cardiovascular phenotypes such as ischemic heart disease, stroke risk, or hypertensive vascular diseases. The blockchain-enabled medical record systems, along with the secure 5G-SDN infrastructure, will guarantee that explainable diagnostic services can avail in distributed healthcare ecosystems. The building of an adaptive AI system in responses to the ever-diversified feedback from patient levels would largely ride on reinforcement learning techniques strategically put in place to optimize model parameters over temporal instance sets. Ultimately, the model’s validation using retinal images acquired from low-cost, portable fundus cameras in under-resourced areas will fast-track AI cardiovascular screening’s global applicability sets.

A key direction for future research is the integration of longitudinal patient data to evaluate the model’s ability to predict future cardiovascular events rather than static risk scores. Additionally, validation within real-world clinical workflows using live patient data will be explored to assess the practical applicability, robustness, and clinical reliability of the proposed system.

### Limitations

Despite demonstrating strong performance, the proposed framework is subject to several limitations. First, model bias may arise due to dependence on manual vessel segmentation annotations, which can introduce variability from human operators. Although the segmentation consistency module mitigates this issue to some extent, future work will explore fully unsupervised or weakly supervised vessel learning approaches to reduce reliance on manual annotations.

Second, the use of synthetically generated clinical metadata based on normal distributions, while useful for controlled experimentation, limits the model’s exposure to real-world clinical heterogeneity. This may affect its generalizability to diverse patient populations. Future research will focus on incorporating real-world clinical datasets to better capture complex patient-specific variations.

Third, the absence of prospectively collected cardiovascular outcome data linked to retinal imaging datasets limits clinical validation of the proposed framework. Future work will address this by validating the model using longitudinal and clinically annotated datasets to improve its real-world applicability.

Additionally, although the model achieves an inference time below 1.5 s per image, the modular architecture may pose challenges for deployment in resource-constrained environments without GPU support. This limitation can be addressed through model compression and hardware-optimized implementations for edge deployment.

Furthermore, the interpretability modules rely on saliency-based methods, which are not yet fully standardized across medical imaging domains and may affect reproducibility. Future efforts will investigate more robust and standardized explainability techniques to enhance consistency and reliability.

Finally, while multiple high-quality datasets were used, limited diversity in ethnicity and geographic representation may restrict the model’s generalizability to global populations. Expanding the training data to include more diverse and representative datasets will be a key direction for future work.

## Data Availability

The datasets used in this study are publicly available and can be accessed from the following sources: MESSIDOR Dataset: [https://www.adcis.net/en/third-party/messidor/]. STARE Dataset: [https://cecas.clemson.edu/~ahoover/stare/]. AV-WIDE Dataset: Publicly available retinal imaging dataset used in prior studies.

## Abbreviations

AUC: Area Under the Curve
AI: Artificial Intelligence
AV: Arteriovenous
AV-WIDE: Arteriovenous-Wide Retinal Imaging Dataset
CNN: Convolutional Neural Network
DNN: Deep Neural Network
DR: Diabetic Retinopathy
eGFR: Estimated Glomerular Filtration Rate
HbA1c: Hemoglobin A1c
HVAAT: Hierarchical Vessel-level Attention Aggregation Transformer
IT: Information Technology
MAE: Mean Absolute Error
MESSIDOR: Méthodes d’Evaluation Subjective Systématique des Images de la Rétine
NHANES: National Health and Nutrition Examination Survey
OCT: Optical Coherence Tomography
PaVSCM: Pathophysiologically Validated Structural Consistency Module
RVO: Retinal Vein Occlusion
STARE-VAS: Structured Texture Analysis and Retinal Evaluation - Vessel Annotation Set
T2DM: Type 2 Diabetes Mellitus
3PM: Predictive, Preventive, and Personalized Medicine
ECG: Electrocardiogram
SBI: Silent Brain Infarction
GCA: Giant Cell Arteritis
CVD: Cardiovascular Disease
VSM: Vascular Structure Modeling
SS-OCTA: Swept-Source Optical Coherence Tomography Angiography
VTA: Vessel Tortuosity Analysis

## References

[1] Hu W, Lin Z, Clark M, Henwood J, Shang X, Chen R, Kiburg K, Zhang L, Ge Z, van Wijngaarden P, Zhu Z, He M. Real-world feasibility, accuracy and acceptability of automated retinal photography and AI-based cardiovascular disease risk assessment in Australian primary care settings: a pragmatic trial. npj Digit Med. 2025;8(1). 10.1038/s41746-025-01436-1. 10.1038/s41746-025-01436-1 PMC11850881 39994433

[2] Diel NJ, Gerner ST, Doeppner TR, Juenemann M, Maxhuni T, Frühwald T, Worm A, Alhaj Omar O, Lytvynchuk L, Struffert T, Bauer P, Huttner HB. Comparison of vascular risk profile and clinical outcomes among patients with central (branch) retinal artery occlusion versus amaurosis fugax. Neurol Res Pract. 2024;6(1). 10.1186/s42466-024-00326-3. 10.1186/s42466-024-00326-3 PMC11097454 38750601

[3] Miao H, Liu S, Wang Z, Ke Y, Cheng L, Yu W, Yu D, Zhang K, Gao Y, Sun Z. Artificial intelligence-derived retinal age gap as a marker for reproductive aging in women. npj Digit Med. 2025;8(1). 10.1038/s41746-025-01699-8. 10.1038/s41746-025-01699-8 PMC12170912 40523954

[4] Yang S, Zhu Z, Chen S, Yuan Y, He M, Wang W. Metabolic fingerprinting on retinal pigment epithelium thickness for individualized risk stratification of type 2 diabetes mellitus. Nat Commun. 2023;14(1). 10.1038/s41467-023-42404-1. 10.1038/s41467-023-42404-1 PMC10585002 37852995

[5] Niu Y, Li X, Guo J, Luo S, Shang X, Liu J, Liu S, He M, Shi D, Huang Y, Zhang H. Comprehensive genome-wide analysis of retinal vessel caliber reveals microvascular-blood pressure pathways: advancing predictive, preventive, and personalized medicine. EPMA J. 2025;16(2):401–17. 10.1007/s13167-025-00411-w. 10.1007/s13167-025-00411-w PMC12106259 40438498

[6] Chen X, Si H, Fu Y, Yang W, Luo Y, Xiao W. Association of retinal microvascular abnormalities with all-cause and specific-cause mortality among U.S. adults. BMC Public Health. 2024;24(1). 10.1186/s12889-024-21117-0. 10.1186/s12889-024-21117-0 PMC11667902 39716194

[7] Fu X, Pan X, Hu Y, Du Z, Zhu Q, Yi Q, Han F. Association between life’s essential 8 cardiovascular health score and glaucoma risk: evidence from NHANES. Int Ophthalmol. 2025;45(1). 10.1007/s10792-025-03620-4. 10.1007/s10792-025-03620-4 PMC12159081 40498226

[8] Germanese C, Anwer A, Eid P, Steinberg L, Guenancia C, Gabrielle P, Creuzot-Garcher C, Meriaudeau F, Arnould L. Artificial intelligence-based prediction of neurocardiovascular risk score from retinal swept-source optical coherence tomography–angiography. Sci Rep. 2024;14(1). 10.1038/s41598-024-78587-w. 10.1038/s41598-024-78587-w PMC11544092 39511360

[9] Courtie E, Taylor M, Danks D, Acharjee A, Jackson T, Logan A, Veenith T, Blanch RJ. Oculomic stratification of COVID-19 patients’ intensive therapy unit admission status and mortality by retinal morphological findings. Sci Rep. 2024;14(1). 10.1038/s41598-024-68543-z. 10.1038/s41598-024-68543-z PMC11393335 39266635

[10] Kha R, Kapucu Y, Indrakumar M, Burlutsky G, Thiagalingam A, Kovoor P, Mitchell P, Liew G. Diabetic retinopathy further increases risk of cardiovascular disease mortality in a high-risk cohort. Sci Rep. 2025;15(1). 10.1038/s41598-025-86559-x. 10.1038/s41598-025-86559-x PMC11808117 39924501

[11] Ari Widjaja S, Mieler WF, Sasono W, Soelistijo SA, Kartasasmita AS, Murakami A, Nakao S. Retinal neurovascular alteration in type 2 diabetes with renal impairment in association with systemic arterial stiffness. Int J Retina Vitreous. 2024;10(1). 10.1186/s40942-023-00521-5. 10.1186/s40942-023-00521-5 PMC10763135 38167275

[12] Lal A, Barry MA, Mitchell P, Thiagalingam A. ECG–gated retinal vessel calibre as a novel measure of aberrant pulsatile retinal flow in diabetes mellitus: a cross-sectional study. J Diabetes Metab Disord. 2024;23(2):1887–98. 10.1007/s40200-024-01439-x. 10.1007/s40200-024-01439-x PMC11599655 39610487

[13] Wang C, Hou J, Lin S, Wang J, Ding J, Liu C, Jiang Z, Bao N. Association between liver fibrosis’s noninvasive scores and retinal imaging changes: insights from NHANES. J Health Popul Nutr. 2025;44(1). 10.1186/s41043-025-00805-6. 10.1186/s41043-025-00805-6 PMC11871793 40022221

[14] Pushpanathan K, Bai Y, Lei X, Goh JHL, Xue CC, Yew SME, Chee M, Quek TC, Peng Q, Soh ZD, Yu MCY, Zhou J, Wang Y, Jonas JB, Wang X, Sim X, Tai ES, Sabanayagam C, Goh RSM, Liu Y, Cheng C, Tham Y. Vision transformer-based stratification of pre/diabetic and pre/hypertensive patients from retinal photographs for 3PM applications. EPMA J. 2025;16(2):519–33. 10.1007/s13167-025-00412-9. 10.1007/s13167-025-00412-9 PMC12106178 40438493

[15] El Husseini N, Schaich CL, Craft S, Rapp SR, Hayden KM, Sharrett R, Cotch MF, Wong TY, Luchsinger JA, Espeland MA, Baker LD, Bertoni AG, Hughes TM. Retinal vessel caliber and cognitive performance: the multi-ethnic study of atherosclerosis (MESA). Sci Rep. 2024;14(1). 10.1038/s41598-024-54412-2. 10.1038/s41598-024-54412-2 PMC10876697 38374377

[16] Lee TK, Kim SY, Choi HJ, Choe EK, Sohn K. Vision transformer based interpretable metabolic syndrome classification using retinal images. npj Digit Med. 2025;8(1). 10.1038/s41746-025-01588-0. 10.1038/s41746-025-01588-0 PMC11992118 40216912

[17] Yu Z, Chen R, Gui P, Wang W, Razzak I, Alinejad-Rokny H, Zeng X, Shang X, Zhang L, Yang X, Yu H, Huang W, Lu H, van Wijngaarden P, He M, Zhu Z, Ge Z. A cross population study of retinal aging biomarkers with longitudinal pre-training and label distribution learning. npj Digit Med. 2025;8(1). 10.1038/s41746-025-01751-7. 10.1038/s41746-025-01751-7 PMC12152120 40494933

[18] Günthner R, Lorenz G, Braunisch MC, Angermann S, Matschkal J, Hausinger R, Kuchler T, Glaser P, Schicktanz F, Haller B, Heemann U, Streese L, Hanssen H, Kotliar K, Schmaderer C. Endothelial dysfunction in retinal vessels of hemodialysis patients compared to healthy controls. Sci Rep. 2024;14(1). 10.1038/s41598-024-64581-9. 10.1038/s41598-024-64581-9 PMC11183144 38886448

[19] Yu NH, Shin D, Ryu IH, Yoo TK, Koh K. Retinal vein occlusion risk prediction without fundus examination using a no-code machine learning tool for tabular data: a nationwide cross-sectional study from South Korea. BMC Med Inform Decis Mak. 2025;25(1). 10.1186/s12911-025-02950-8. 10.1186/s12911-025-02950-8 PMC11889835 40055729

[20] Koseoglu ND, Wang J, Anokye-Danso F, Amezcua Moreno J, Cha E, Fuchs F, Teed J, Yao J, Zhang Y, Ahima RS, Sachdeva MM. Association of serum adiponectin and leptin levels with inner retinal thickness among individuals with or without elevated HbA1c. Sci Rep. 2025;15(1). 10.1038/s41598-025-93562-9. 10.1038/s41598-025-93562-9 PMC11904190 40075217

[21] Fathimah FSN, Widjaja A, Sasono S, Yustiarini W, Firmansjah I, Prakosa M, Mulyazhara AD, A. K., Soelistijo SA. Retinal vessel tortuosity and fractal dimension in diabetic retinopathy. Int J Retina Vitreous. 2025;11(1). 10.1186/s40942-025-00688-z. 10.1186/s40942-025-00688-z PMC12164056 40506774

[22] Mustafar R, Hishamuddin KAM, Mohd R, Kamaruzaman L, Halim WHWA, Hsien YM, Sze TK, Zaki WMDW, Ali A, Bain A. Retinal changes and cardiac biomarker assessment in relation to chronic kidney disease: a single centre study. BMC Nephrol. 2023;24(1). 10.1186/s12882-023-03386-w. 10.1186/s12882-023-03386-w PMC10644488 37957551

[23] Jain PK, Tadepalli KV, Roy S, Sharma N. Exploring deep learning for carotid artery plaque segmentation: atherosclerosis to cardiovascular risk biomarkers. Multimed Tools Appl. 2023;83(14):42765–97. 10.1007/s11042-023-17243-3.

[24] Zhou Y, Chia MA, Wagner SK, Ayhan MS, Williamson DJ, Struyven RR, Liu T, Xu M, Lozano MG, Woodward-Court P, Kihara Y, Allen N, Gallacher JEJ, Littlejohns T, Aslam T, Bishop P, Black G, Sergouniotis P, Atan D, Dick AD, Williams C, Barman S, Barrett JH, Mackie S, Braithwaite T, Carare RO, Ennis S, Gibson J, Lotery AJ, Self J, Chakravarthy U, Hogg RE, Paterson E, Woodside J, Peto T, Mckay G, Mcguinness B, Foster PJ, Balaskas K, Khawaja AP, Pontikos N, Rahi JS, Lascaratos G, Patel PJ, Chan M, Chua SYL, Day A, Desai P, Egan C, Fruttiger M, Garway-Heath DF, Hardcastle A, Khaw SPT, Moore T, Sivaprasad S, Strouthidis N, Thomas D, Tufail A, Viswanathan AC, Dhillon B, Macgillivray T, Sudlow C, Vitart V, Doney A, Trucco E, Guggeinheim JA, Morgan JE, Hammond CJ, Williams K, Hysi P, Harding SP, Zheng Y, Luben R, Luthert P, Sun Z, McKibbin M, O’Sullivan E, Oram R, Weedon M, Owen CG, Rudnicka AR, Sattar N, Steel D, Stratton I, Tapp R, Yates MM, Petzold A, Madhusudhan S, Altmann A, Lee AY, Topol EJ, Denniston AK, Alexander DC, Keane PA. A foundation model for generalizable disease detection from retinal images. Nature. 2023;622(7981):156–163. 10.1038/s41586-023-06555-x. 10.1038/s41586-023-06555-x PMC10550819 37704728

[25] Farrah TE, Pugh D, Chapman FA, Godden E, Balmforth C, Oniscu GC, Webb DJ, Dhillon B, Dear JW, Bailey MA, Gallacher PJ, Dhaun N. Choroidal and retinal thinning in chronic kidney disease independently associate with eGFR decline and are modifiable with treatment. Nat Commun. 2023;14(1). 10.1038/s41467-023-43125-1. 10.1038/s41467-023-43125-1 PMC10697963 38052813

[26] Jiang N, Ji H, Guan Z, Pan Y, Deng C, Guo Y, Liu D, Chen T, Wang S, Wu Y, Yang D, Ran AR, Hamzah H, Chee ML, Yin C, Thinggaard BS, Pedersen FN, Peng Q, Quek TC, Goh JHL, Singh S, Abd Raof AS, Lee-Boey JWS, Lu Y, Huang S, Shu J, Yu S, Jin Y, Li T, Qin Y, Wang J, Yang X, Hu T, Wang Z, Zhao Y, Lee S, Wei X, Zheng H, Li Y, Shen J, Zhou Y, Lin S, Wu C, Dai R, Ruan L, Hogg RE, Wright D, Wang YX, Zheng Y, Tan GSW, Sabanayagam C, Bao Y, Zhang C, Zhang P, Zou W, Guo M, Yang X, McKay GJ, Grauslund J, Lim L, Li Z, Cheung CY, Tham YC, Cheng C, Wang Y, Dai Q, Jia W, Li H, Sheng B, Wong TY. A deep learning system for detecting silent brain infarction and predicting stroke risk. Nat Biomed Eng. 2025. 10.1038/s41551-025-01413-9. 10.1038/s41551-025-01413-9 PMC12623247 40481238

[27] S B, Kadry S, Dhanaraj RK, K SK, Manthiramoorthy C. Res-Unet based blood vessel segmentation and cardiovascular disease prediction using chronological chef-based optimization algorithm based deep residual network from retinal fundus images. Multimed Tools Appl. 2024;83(40):87929–58. 10.1007/s11042-024-18810-y.

[28] Nabrdalik K, Irlik K, Meng Y, Kwiendacz H, Piaśnik J, Hendel M, Ignacy P, Kulpa J, Kegler K, Herba M, Boczek S, Hashim EB, Gao Z, Gumprecht J, Zheng Y, Lip GYH, Alam U. Artificial intelligence-based classification of cardiac autonomic neuropathy from retinal fundus images in patients with diabetes: The Silesia Diabetes Heart Study. Cardiovasc Diabetol. 2024;23(1). 10.1186/s12933-024-02367-z. 10.1186/s12933-024-02367-z PMC11316981 39127709

[29] Chen S, Xu Y, Chen B, Lin S, Lu L, Cheng M, Wang Y, Yang Q, Ling S, Zhou D, Shi Y, Zou H, Ma Y. Remnant cholesterol is correlated with retinal vascular morphology and diabetic retinopathy in type 2 diabetes mellitus: a cross-sectional study. Lipids Health Dis. 2024;23(1). 10.1186/s12944-024-02064-6. 10.1186/s12944-024-02064-6 PMC10926603 38468242

[30] Putera I, Quiros JDV, Rombach SM, Dik WA, van Hagen PM, La Distia Nora R. Artificial intelligence-based uveitis diagnosis through retinal vasculature analysis: a paradigm shift in ocular tuberculosis. Ophthalmol Ther. 2025;14(4):717–32. 10.1007/s40123-025-01103-4. 10.1007/s40123-025-01103-4 PMC11920457 39992617

[31] Dinah C, Chang A, Lee J, Li WW, Singh R, Wu L, Wong D, Saffar I. What is occluding our understanding of retinal vein occlusion? Ophthalmol Ther. 2024;13(12):3025–34. 10.1007/s40123-024-01042-6. 10.1007/s40123-024-01042-6 PMC11564720 39387960

[32] Quaranta L, Galbussera AA, Tettamanti M, Novella A, Pasina L, Fortino I, Leoni O, Oddone F, Giammaria S, Kużniak M, Weinreb RN, Nobili A. Relationships among glaucoma, cardiovascular diseases, and mortality. Adv Ther. 2025. 10.1007/s12325-025-03282-9. 10.1007/s12325-025-03282-9 PMC12394243 40608283

[33] Sojo Vega L, Recasens M, Martínez J, Aguilera A, Ayala M, Admetlla N, Pellicer P, Blay C, Fabregat B, Esteve-Serra M, Riera L, Barahona R, Xifra G, Esteve E, Biarnés J, Pérez D, Gifre G, Mauri S, Costa E, Wos M, Buxó M, Fernández-Balsells M. Unseen threat: how subclinical atherosclerosis increases mortality risk in patients with type 1 diabetes. Cardiovasc Diabetol. 2024;23(1). 10.1186/s12933-024-02455-0. 10.1186/s12933-024-02455-0 PMC11488122 39420367

[34] Muthukumar KA, Nandi D, Ranjan P, Ramachandran K, PJ S, Ghosh A, M A, Radhakrishnan A, Dhandapani VE, Janardhanan R. Integrating electrocardiogram and fundus images for early detection of cardiovascular diseases. Sci Rep. 2025;15(1). 10.1038/s41598-025-87634-z. 10.1038/s41598-025-87634-z PMC11799439 39910082

[35] Cai B, Zhou Y, Yang X, Wang Z, Huang C, Xiao Q, Jiang H, Zhao Y, Tian X, Wang Q, Li G, Li M, Zeng X, Zhao J. Remnant cholesterol predicts risk of recurrent thrombosis beyond LDL-cholesterol in patients with antiphospholipid syndrome. BMC Med. 2025;23(1). 10.1186/s12916-025-04063-5. 10.1186/s12916-025-04063-5 PMC12016284 40264203

[36] El Miedany Y, El Gaafary M, Toth M, Abdel Azim A, Palmer D, Dolbear G, Affam D, Hassan W, Tabra S, Saber S, Abu-Zaid M. Step forward towards treat-to-target management of giant cell arteritis: patients stratification aiming to targeted remission – updated guidelines. Egypt Rheumatol Rehabil. 2024;51(1). 10.1186/s43166-024-00237-w.

[37] Huang Y, Syed MG, Chen R, Li C, Shang X, Wang W, Zhang X, Zhang X, Tang S, Liu J, Liu S, Srinivasan S, Hu Y, Mookiah MRK, Wang H, Trucco E, Yu H, Palmer C, Zhu Z, Doney ASF, He M. Genomic determinants of biological age estimated by deep learning applied to retinal images. GeroScience. 2025;47(2):2613–29. 10.1007/s11357-024-01481-w. 39775603 10.1007/s11357-024-01481-w PMC11979078

[38] Sala Vila A, Vinagre I, Cofán M, Lázaro I, Alé-Chilet A, Barraso M, Hernandez T, Harris WS, Zarranz Ventura J, Ortega E. Blood omega-3 biomarkers, diabetic retinopathy and retinal vessel status in patients with type 1 diabetes. Eye. 2025;39(8):1526–31. 10.1038/s41433-025-03705-5. 10.1038/s41433-025-03705-5 PMC12089322 39966603

[39] Ueyama C, Horibe H, Maekawa Y, Hiramatsu S, Yamase Y, Funabiki J, Takemoto Y, Shigeta T, Hibino T, Kondo T, Yatsuya H, Ishii H, Murohara T. Relationship between abdominal visceral adipose tissue and cardiovascular events in patients with acute coronary syndrome. Heart Vessels. 2025. 10.1007/s00380-025-02557-z. 40418252 10.1007/s00380-025-02557-z

[40] Malik R, Beaufort N, Li J, et al. Genetically proxied HTRA1 protease activity and circulating levels independently predict risk of ischemic stroke and coronary artery disease. Nat Cardiovasc Res. 2024;3:701–13. 10.1038/s44161-024-00475-3. 39196222 10.1038/s44161-024-00475-3

